# Phase I clinical study of multiple epitope peptide vaccine combined with chemoradiation therapy in esophageal cancer patients

**DOI:** 10.1186/1479-5876-12-84

**Published:** 2014-04-03

**Authors:** Hisae Iinuma, Ryoji Fukushima, Tsuyoshi Inaba, Junko Tamura, Taisuke Inoue, Etsushi Ogawa, Masahiro Horikawa, Yoshibumi Ikeda, Noriyuki Matsutani, Kazuyoshi Takeda, Koji Yoshida, Takuya Tsunoda, Tadashi Ikeda, Yusuke Nakamura, Kota Okinaga

**Affiliations:** 1 Department of Surgery, Teikyo University School of Medicine, Tokyo, Japan; 2 Laboratory of Molecular Medicine, Human Genome Center, Institute of Medical Science, University of Tokyo, Tokyo, Japan; 3 OncoTherapy Science Incorporation, Research and Development Division, Kanagawa, Japan; 4 The University of Chicago, Chicago, IL, USA; 5 Department of Immunology, Juntendo University School of Medicine, Tokyo, Japan

**Keywords:** Cancer vaccine, Chemoradiation therapy, Esophageal cancer, CTL, Phase I clinical trial

## Abstract

**Background:**

Chemoradiation therapy (CRT) has been widely used for unresectable esophageal squamous cell carcinoma (ESCC) patients. However, many patients develop local recurrence after CRT. In this study, we hypothesized that the immunotherapy by peptide vaccine may be effective for the eradication of minimal residual cancer cells after CRT. This study was conducted as a phase I clinical trial of multiple-peptide vaccine therapy combined with CRT on patients with unresectable ESCC.

**Patients and methods:**

HLA-A*2402 positive 11 unresectable chemo-naïve ESCC patients were treated by HLA-A*2402-restricted multi-peptide vaccine combined with CRT. The peptide vaccine included the 5 peptides as follows; TTK protein kinase (TTK), up-regulated lung cancer 10 (URLC10), insulin-like growth factor–II mRNA binding protein 3 (KOC1), vascular endothelial growth factor receptor 1 (VEGFR1) and 2 (VEGFR2). CRT consisted of radiotherapy (60 Gy) with concurrent cisplatin (40 mg/m^2^) and 5-fluorouracil (400 mg/m^2^). Peptide vaccines mixed with incomplete Freund’s adjuvant were injected subcutaneously once a week on at least 8 occasions combined with CRT.

**Results:**

Vaccination with CRT therapy was well-tolerated, and no severe adverse effects were observed. In the case of grade 3 toxicities, leucopenia, neutropenia, anemia and thrombocutopenia occurred in 54.5%, 27.3%, 27.3% and 9.1% of patients, respectively. Grade 1 local skin reactions in the injection sites of vaccination were observed in 81.8% of patients. The expressions of HLA class I, URLC10, TTK, KOC1, VEGFR1 and VEGFR2 antigens were observed in the tumor tissues of all patients. All patients showed peptide-specific cytotoxic T lymphocytes responses in at least one of the 5 kinds of peptide antigens during the vaccination. Six cases of complete response (CR) and 5 cases of progressive disease (PD) were observed after the 8^th^ vaccination. The 4 CR patients who continued the peptide vaccination experienced long consistent CR for 2.0, 2.9 4.5 and 4.6 years.

**Conclusions:**

A combination therapy of multi-peptide vaccine with CRT can successfully be performed with satisfactory levels of safety, and application of this combination therapy may be an effective treatment for patients with unresectable ESCC.

**Trial registration:**

ClinicalTrial.gov, number NCT00632333.

## Background

Esophageal squamous cell carcinoma (ESCC) is a highly malignant disease, especially in Asia. In Japan, the number of deaths attributable to ESCC has been slowly increasing, and 11,345 people died in 2011 [1]. Recent developments in surgical techniques and postoperative management including chemotherapy and fractionized radiation therapy, have contributed to improvements in the surgical outcome [2,3]. Chemoradiation therapy (CRT) has been used in Japan since 1990, especially for unresectable ESCC patients with locally advanced disease and/or distant metastasis, or for those who were not fit to undergo surgery [3]. However, it has been reported that many patients develop local recurrence soon after CRT [4-8]. This recurrence is due to the difficulty in making a precise clinical assessment of CRT on the treated cancer tissues. Viable cancer cells are thought to remain at the primary site in the majority of patients even if clinical complete response (CR) is accomplished. Although salvage esophagectomy was recommended in recurrent patients after CRT, high incidences of mortality and radiation-related post-operative complications, such as pneumonitis and cardiomyositis, have been reported [9]. Therefore, the development of a new approach for residual cancer cells after CRT is necessary to improve the prognosis of patients with unresectable ESCC.

A multimodality approach for ESCC is preferred in order to improve prognosis of CRT, and immunotherapy can be viewed as one rational approach for combination therapy with CRT. Recent studies have suggested that local irradiation elicits immunomodulatory effects and induces tumor-specific immune responses [10-14]. Furthermore, fluorouracil (5-FU) and cisplatin (CDDP), the standard agents for the treatment of ESCC, may immunomodulate the anti-tumor immunological response through the down regulation of immunosuppressive regulatory T cells and/ or increase the expression of MHC molecules [15-17]. In this study, we hypothesized that the CRT may act immunogenically, and immunotherapy with peptide vaccine may be effective for the eradication of residual cancer cells after CRT.

By using cDNA microarray technology coupled with laser microdisection, we identified novel HLA-A*2402 (which is the most common HLA-A allele in the Japanese population) - restricted epitope peptides as targets for cancer vaccination [18-21]. In particular, it has been demonstrated that TTK protein kinase (TTK), up-regulated lung cancer 10 (URLC10) and insulin-like growth factor–II mRNA binding protein 3 (KOC1) are promising targets for cancer vaccination in advanced ESCC patients [22,23]. Furthermore, to overcome the inhibition of the antitumor effects of cytotoxic T lymphocytes (CTL), which occur due to the down regulation of human leukocyte antigen (HLA), we focused on the peptide vaccine targeting the vascular endothelial growth factor receptor 1 (VEGFR1) and 2 (VEGFR2). It has been reported that CTLs were strongly induced by these peptides in various cancer patients [24].

In this study, we attempted a phase I clinical trial of multi-peptide vaccines in combination with CRT for unresectable ESCC patients. We selected 5 peptide vaccines (TTK, URLC10, KOC1, VEGFR1 and VEGFR2) to overcome the immune-escape mechanisms and increase the therapeutic potential of the cancer vaccine. The primary endpoint of this study is an evaluation of the safety factors, and secondary endpoints are estimation of the immune responses and clinical responses.

## Materials and methods

### Study design and treatment protocol

This study was a phase I clinical trial of dose-escalated multiple-peptide vaccine (TTK, URLC10, KOC1, VEGFR1 and VEGFR2) combined with CRT. Each peptide vaccine was mixed with incomplete Freund’s adjuvant (IFA) (Montanide ISA 51; Seppic, Paris, France) separately, and was injected subcutaneously in 11 unresectable ESCC patients with stage II (3 cases), stage III (4 cases) or stage IV (4 cases). All patients were chemo-naïve. Treatment consisted of 2 courses of CDDP 40 mg/m^2^ (day1, 8) and 5-FU 400 mg/m2/day (days 1-5, 8-12) combined with concurrent radiotherapy of 60 Gy in 30 fractions as described previously [7]. During the break period of chemotherapy, 5 kinds of peptide vaccines were administered once every week, a total 8 times (Additional file 1: Figure S1). Dose escalation was performed in 3 patients’ cohort with dose of 0.5 mg, 1 mg and 3 mg for each peptide. For the imaging analysis, computed tomography (CT) scan and endoscopy were performed at pre-treatment period and every 2 ~ 3 months after the vaccination, and every measureable legion was evaluated by RECIST. CR patients additionally received 5 kinds of peptide vaccine, once every 2 weeks for 1 year and then once a month until changes to the progressive disease (PD) were observed. The primary endpoint of this trial was to evaluate the safety of this therapy. The secondary endpoints were to investigate the immunological response and clinical outcome. This study was approved by the Ethics Committee on Clinical Investigation of Teikyo University (Tokyo, Japan) and is registered with Clinical Trials.gov (NCT 00632333). Written informed consent was obtained from all individuals. The trial was carried out in accordance with the Helsinki declaration on experimentation on human subjects.

### Patients eligibility

The eligibility criteria for patients participating in the clinical trial were as follows: (1) they were unresectable ESCC patients with widespread ESCC who refused surgical resection, locally advanced or metastatic disease; (2) they have disease which is possible the evaluation of clinical response ( no limitation with respect to the presence or absence of measurable diseases according to RECIST ); (3) they were HLA-A*2402-positive patients by DNA typing of HLA-A genetic variations; (4) no therapy 4 weeks prior to the initiation of the trial; (5) ECOG performance status was 0-2; (6) their expected survival was at least 3 months; (7) the age of the patients was between >20 and <80 years; and (8) adequate bone-marrow, cardiac, pulmonary, hepatic and renal functions had to be present, including white blood cell count >2000/mm^3^, platelet count >75000/mm^3^, total bilirubin < 1.5 times of the institutional normal upper limits, creatinine < 1.5 times of the institutional normal upper limits, AST/ALT/ALP <2.5 times of the institutional normal upper limits. The exclusion criteria of patients participating in the clinical trial were as follows. (1) pregnancy; (2) breastfeeding; (3) active or uncontrolled infection; (4) concomitant treatment with steroids or immunosuppresing agents; (5) other active or uncontrolled other malignancy; (6) disease to the central nervous system; (7) some other form of unsuitableness as determined by the principal investigator or physician.

### Toxicity assessment

Signs of toxicities were assessed by the Common Terminology Criteria for Adverse Event (CTCAE) version 3. Blood count and serum chemistry tests were performed every 2 weeks. The worst toxicity throughout the treatment period (2 courses of CRT and 8^th^ peptide vaccination) was investigated. Dose escalation was performed in 3 patients’ cohort with dose of 0.5 mg, 1 mg and 3 mg for each peptide. Dose-limiting toxicity (DLT) was defined as a hematological toxicity of grade 4 and non-hematological toxicity of grade 3 or greater.

### Peptides

The peptides derived from TTK-567 (SYRNEIAYL), URLC10-177 (RYCNLEGPPI), KOC1-508 (KTVNELQNL), VEGFR1-1084 (SYGVLLWEIF), VEGFR2-169 (RFVPDGNRI) that bound to the HLA-A*24 molecule were synthesized by the American Peptide Company (Sunnyvale, CA, USA). The purity (>97%) and identify of the peptides were determined by analytical high-performance liquid chromatography (HPLC) and mass spectrometry analysis, respectively. The endotoxin levels and bioburden of these peptides were tested and determined to be within acceptable levels as GMP grade for the vaccines (NeoMPS, Inc.).

### Measurement of CTL responses

An enzyme-linked immunospot (ELISPOT) assay was performed to measure the peptide specific CTL response, as described previously [23,25]. For the evaluation of CTL and Flow cytometry, blood samples were obtained from the patients at the pre-vaccination period and after the 4^th^, 8^th^, 12^th^ and 16^th^ vaccinations. Briefly, peripheral blood mononuclear cells (PBMCs) of blood samples were cultured with respective peptide and IL-2 (Novartis, Emeryville, CA) at 37°C for two weeks. Peptide was added into the culture at day 0 and day 7. Following CD4^+^ cell depletion by Dynal CD4 positive isolation kit (Invitrogen, Carlsbad, CA), IFN-γ ELISPOT assay was performed using Human IFN-γ ELISPOT PLUS kit (MabTech, Nacka Strand, Sweden) according to the manufacturer’s instructions. The number of peptide specific spots was calculated by subtracting the spot number in the control well from the spot number of wells with peptide-pulsed TISI cells. The positivity of the antigen-specific T cell responses were classified into four grades (-, +, ++ and +++), depending on the peptide-specific spots at different responder/stimulator ratios. When the algorithm indicated +, ++ or +++, we judged it to be a positive case.

### Flow cytometry

Expression of peptide specific T cell receptors was analyzed on FACS-CantoII (Becton Dickinson, San Jose, CA) using URLC10, TTK, KOC1, VEGFR1 or VEGFR2-derived epitope peptide-MHC dextramer (Immudex, Copenhagen, Denmark) or tetramer-PE (Medical & Biological Laboratories Co.,Ltd., Nagoya, Japan) according to the manufacturer’s instructions. HIV-derived epitope peptide (RYLRDQQLL)-MHC dextramer or tetramer-PE was used as a negative control. T cells were incubated with peptide-MHC dextramer or tetramer-PE, treated with FITC-conjugated anti-human CD8 mAb, APC-conjugated anti-human CD3 mAb, PE-Cy7-conjugated anti-human CD4 mAb, and 7-AAD (BD Pharmingen, San Diego, CA.), and then analyzed with flow cytometry.

### Immunohistchemical

Immunohistochemical (IHC) staining of HLA class I, TTK, URLC10, KOC1, VEGFR1 and VEGFR2 antigens of the ESCC and adjacent normal tissues were investigated using the serial sections of formalin-fixed, paraffin-embedded biopsy samples, as described previously [25]. The primary antibodies used in this study were as follows: HLA class I (Cosmo Bio. Co.Ltd. Tokyo, Japan), URLC10 (Imagenex, San Diego, CA.), TTK (Novus Bio. LLC. Littleton, CO.), KOC1 (Santacruz Bio. Inc. Dallas, Texas), VEGFR1 (Novus Bio. LLC.) and VEGFR2 (R&D Systems Inc., Minneapolis, MN). Immunoreaction was detected using the following secondary antibody systems: Simple Stain MAX-PO kit (Nichirei Bioscience, Tokyo, Japan) for HLA class I, URLC10, KOC1 and TTK; and CSA-II Biotin-free Tyramide Single Amplification System (Dako Inc., Carpinteria, CA) for VEGFR1 and VEGFR2, according to the instructions of the manufacturer. The intensity of staining was evaluated using the following criteria: strong positive staining of more than 80% (+++), positive staining of 50-80% (++); positive less than 50% (+); and no appreciable staining in tumor cells (-).

### Statistical analysis

For the statistical analysis of data, JMP v9 software was used (SAS Inc.).

## Results

### Patients’ characteristics

Between March 2008 and October 2011, 11 unresectable ESCC patients enrolled in this study. The characteristics of these patients are shown in Table 1. The patients comprised 10 males and 1 female, with a median age of 65 years (range 58-71 years). All patients were positive for HLA-A*2402. Performance status was 0 for 8 patients, 1 for 1 patient and 2 for 2 patients. The stages of ESCC were: 3 patients with stage II (No 1, 2, 7), 4 patients with stage III (No.4, 5, 6, 10) and 4 patients with stage IV (No. 3, 8, 9, 11). These patients were unresectable by reason of widespread ESCC (No 1, 2, 7), lymph node metastasis (No 2-11), adrenal gland metastasis (No.9) and lung metastasis (No 11).

**Table 1:** Patients’ characteristics

**Patient no.**	**Dose of peptides (mg)**	**HLA**	**Age (Y)**	**Gender**	Stage*	PS**	**Target organ**
1	0.5	A2402	59	M	IIA	0	Primary tumor
2	0.5	A2402	58	M	IIB	0	Primary tumor
							Lymph node
3	0.5	A2402	69	F	IVA	1	Primary tumor
							Lymph node
4	1.0	A2402	64	M	III	2	Primary tumor
							Lymph node
5	1.0	A2402	65	M	III	2	Primary tumor
							Lymph node
6	1.0	A2402	61	M	III	0	Primary tumor
							Lymph node
7	3.0	A2402	70	M	IIB	0	Primary tumor
							Lymph node
8	3.0	A2402	63	M	IVA	0	Primary tumor
							Lymph node
9	3.0	A2402	67	M	IVB	0	Primary tumor
							Lymph node
							Adrenal gland
10	3.0	A2402	71	M	III	0	Primary tumor
							Lymph node
11	3.0	A2402	67	M	IVB	0	Primary tumor
							Lymph node
							Lung

*Stage according to the TNM classification for esophageal cancer (UICC).

**PS: Performance status (ECOG).

### Adverse events

DLT of our study was evaluated by the dose escalation schedule. The worst toxicity throughout the treatment of 2 courses of CRT and 8^th^ vaccination was investigated in each patient (Table 2). This therapy was well-tolerated without any serious adverse events. Major toxicities were myelo-suppression and esophagus-related toxicities. Grade 3 toxicities of leucopenia, neutropenia, anemia and thrombocytopenia occurred in 54.5%, 27.3%, 27.3% and 9.1% of patients, respectively. Grade 2 toxicities of esophagitis, nausea/vomiting, diarrhea, stomatitis/pharyngitis and hypernatremia occurred in 18.2%, 18.2%, 9.1%, 27.3% and 27.3% of patients, respectively. Grade 1 local skin reactions in the peptide vaccine injection sites were observed in 81.8%. DLT of peptide vaccine was not observed in this trial.

**Table 2:** Summary of adverse events

**Toxicity**	**Peptide**									**Total patients (n = 11)**		
	**0.5 mg (n = 3)**			**1.0 mg (n = 3)**			**3.0 mg (n = 5)**					
	Grade*			**Grade**			**Grade**			**Grade (%)**		
	**1**	**2**	**3**	**1**	**2**	**3**	**1**	**2**	**3**	**1**	**2**	**3**
Dermatology/skin												
Reaction at the injection site	2	0	0	3	0	0	4	0	0	9 (81.8)	0 (0.0)	0 (0.0)
Hematological												
Leukocyte	0	0	2	0	0	2	0	1	2	0 (0.0)	1 (9.1)	6 (54.5)
Neutrocyte	0	1	1	0	1	1	0	2	1	0 (0.0)	4 (36.4)	3 (27.3)
Hemoglobin	1	1	1	1	1	1	1	1	1	3 (27.3)	3 (27.3)	3 (27.3)
Platelet	1	0	0	0	0	1	3	0	0	4 (36.4)	0 (0.0)	1 (9.1)
Non-hematological												
Esophagitis	0	1	0	2	0	0	2	1	0	4 (36.4)	2 (18.2)	0 (0.0)
AST	1	0	0	1	0	0	0	0	0	2 (18.2)	0 (0.0)	0 (0.0)
ALT	0	0	0	0	0	0	1	0	0	1 (9.1)	0 (0.0)	0 (0.0)
Creatinine	0	0	0	0	0	0	0	0	0	0 (0.0)	0 (0.0)	0 (0.0)
Nausea/vomiting	1	0	0	0	1	0	0	0	0	1 (9.1)	2 (18.2)	0 (0.0)
Diarrhea	0	1	0	0	0	0	0	0	0	0 (0.0)	1 (9.1)	0 (0.0)
Stomatitis/pharyngitis	0	1	0	0	1	0	0	1	0	0 (0.0)	3 (27.3)	0 (0.0)
Hyponatremia	0	2	0	1	0	0	1	1	0	2 (18.2)	3 (27.3)	0 (0.0)

*Adverse events were graded according to the CTCAE v3.0.

### Immunohistochemical study

**Figure 1:**
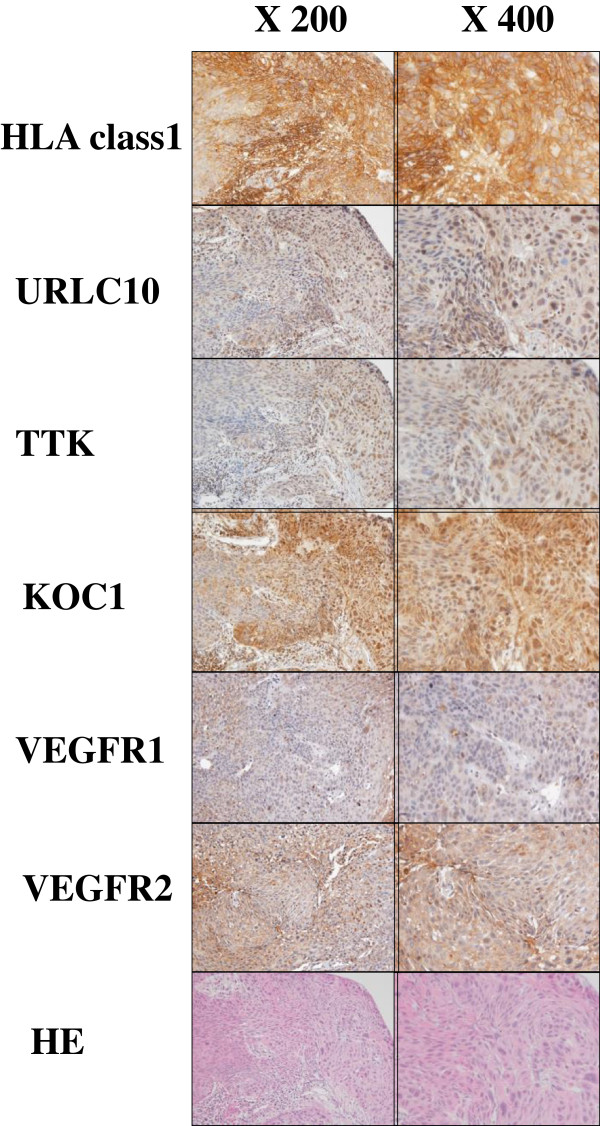
**Immunohistochemical staining of HLA class I, URLC10, TTK, KOC1, VEGFR1 and VEGFR2 antigens in the ESCC tissues.** Typical expression of HLA class I, URLC10, TTK, KOC1, VEGFR1 and VEGFR2 in the primary ESCC tissues obtained from case 6 are shown. The staining was undertaken using the serial sections of the biopsy sample.

We evaluated the expression levels of HLA Class I, URLC10, TTK, KOC1, VEGFR1 and VEGFR2 antigens in biopsy samples of the enrolled patients (Table 3). HLA class I antigen was strong expressed in cell membrane of tumor cells of all cases. The strong expressions of URLC10, TTK and KOC1 antigens were observed in cytoplasm of tumor cells of all patients. The expressions of VEGFR1 and VEGFR2 were observed in tumor cells and stromal vessels of the ESCC tissues of all patients. Representative IHC staining of these antigens in case 6 is shown in Figure 1. Strong expressions of URLC10, TTK, KOC1 and MHC class I antigens were observed in the tumor cells. Expressions of approximately the same intensity of VEGFR1 and VEGFR2 were observed in the tumor cells and stromal vessels of the ESCC tissues.

**Table 3:** Immunohistochemical staining to each antigen of ESCC tissues

**Patients no.**	Immunohistochemistry*					
	**HLA class I**	**URLC10**	**TTK**	**KOC1**	**VEGFR1**	**VEGFR2**
1	+ + +	+ + +	+ + +	+ + +	+ +	+
2	+ + +	+ + +	+ + +	+ + +	+ +	+ +
3	+ + +	+ + +	+ +	+ + +	+	+
4	+ + +	+ + +	+ +	+ + +	+ +	+ +
5	+ + +	+ + +	+ + +	+ + +	+ +	+
6	+ + +	+ + +	+ + +	+ + +	+	+ +
7	+ + +	+ + +	+ + +	+ + +	+ +	+ +
8	+ + +	+ + +	+ +	+ + +	+	+
9	+ + +	+ + +	+ + +	+ + +	+ +	+ +
10	+ + +	+ + +	+ + +	+ + +	+	+
11	+ + +	+ + +	+ + +	+ + +	+ +	+ +

*(+++): strong positive staining of more than 80%.

(++): positive staining of 50-80%, (+): positive less than 50%.

(-): no appreciable staining.

In the adjacent normal esophageal tissues, no staining of these antigens was observed (data not shown).

### Peptide-specific CTL responses

An IFN-γ ELISPOT assay was evaluated using PBMC periodically obtained from patients to assess the cellular immune responses to 5 kinds of peptides. Additional file 2: Table S1 shows the CTL responses to each peptide before and after the 4^th^, 8^th^, 12^th^ and 16^th^ vaccination, and Table 4 shows a summary of the CTL positive rates at each vaccination point. All patients showed peptide-specific CTL responses in at least one of the 5 kinds of peptide antigens during the vaccination. In these peptides, URLC10 showed the highest CTL positive rates. They were 70.0% at the 4^th^ vaccination point, 54.5% at the 8^th^ vaccination point, and 100% at the 12^th^ and 16^th^ vaccination points. CTL positive rates of the other peptides after vaccination, were 27.3- 40.0% for TTK, 45.5-60% for KOC1, 33.3-60% for VEGFR1, and 36.4-80.0% for VEGFR2, respectively. Furthermore, the average peptide numbers per patient which induced CTL on 8^th^ vaccinations were 0.7 in 0.5 mg, 1.3 in 1.0 mg and 3.4 in 3.0 mg peptide administration (Table 5). Three mg of peptide induced peptide-specific CTLs more effectively than the 0.5 mg and 1 mg.

**Table 4:** Summary of positive rates of CTL responses to each peptide antigen

**Positive rates of CTL responses to each antigen in all patients (n = 11)**						
**Number of vaccination**	**URLC10 (%)**	**TTK (%)**	**KOC1 (%)**	**VEGFR1 (%)**	**VEGFR2 (%)**	**CMV (%)**
Pre-vac.	27.3 (3/11)	36.4 (4/11)	40.0 (4/10)	20.0 (2/10)	40.0 (4/10)	100.0 (10/10)
4	70.0 (7/10)	27.3 (3/11)	45.5 (5/11)	33.3 (3/9)	45.5 (5/11)	100.0 (11/11)
8	54.5 (6/11)	36.4 (4/11)	45.5 (5/11)	45.5 (5/11)	36.4 (4/11)	100.0 (11/11)
12	100.0 (5/5)	40.0 (2/5)	60.0 (3/5)	40.0 (2/5)	80.0 (4/5)	100.0 ( 5/ 5)
16	100.0 (5/5)	40.0 (2/5)	60.0 (3/5)	60.0 (3/5)	80.0 (4/5)	100.0 ( 5/ 5)

CTL positive rates on the pre-vaccination, 4^th^, 8^th^, 12^th^ or 16^th^ vaccination were examined in all patients (n = 11), CR patients (n = 6) and PD patients (n = 5). When the ELISPOT Assay indicated +, ++ or +++, we judged it to be positive case. *p values.

**Table 5:** **Number of peptides induced CTL on 8**^**th **^**vaccination**

**Patients no.**	**Dose of peptide (mg)**	**Number of peptides induced CTL**	**Average number of peptides per patient**
1	0.5	0	0.7
2	0.5	2	
3	0.5	0	
4	1.0	1	1.3
5	1.0	1	
6	1.0	2	
7	3.0	1	3.4
8	3.0	3	
9	3.0	4	
10	3.0	5	
11	3.0	4	

*Average number of peptides per patient which induced.

CTL on 8^th^ vaccination.

**Figure 2:**
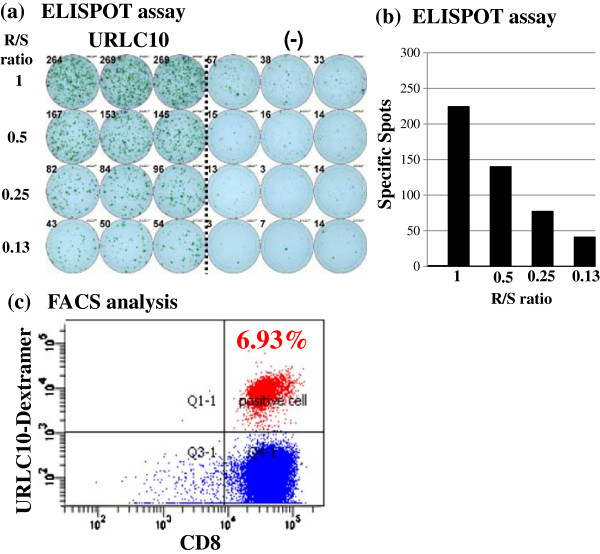
**Representative immunological monitoring assays detecting peptide-specific CTL response.** PBLs obtained from CR patient (case 6) after the 8^th^ vaccination were cultured with URLC10-peptide stimulation and subjected to the ELISPOT assay **(a, b)**. The cultured lymphocytes were analyzed by flow cytometry, and proportion of URLC10 dextramer positive cells in CD8^+^CD3^+^CD4^-^ cells were calculated **(c)**.

Figure 2 shows the representative data from ELISPOT assays against URLC10 in case 6 at the 8^th^ vaccination point. URLC10-specific T cell responses were observed in PBMC derived from patient (Figure 2a, b). Moreover, the peptide-specific CD8 (+) T cells in the cultured T cells were confirmed by the flow cytometry, and 6.93% URLC10 dextramer positive cells were confirmed in the CD8^+^CD3^+^CD4^-^ T cells (Figure 2c).

### Clinical outcome

**Figure 3:**
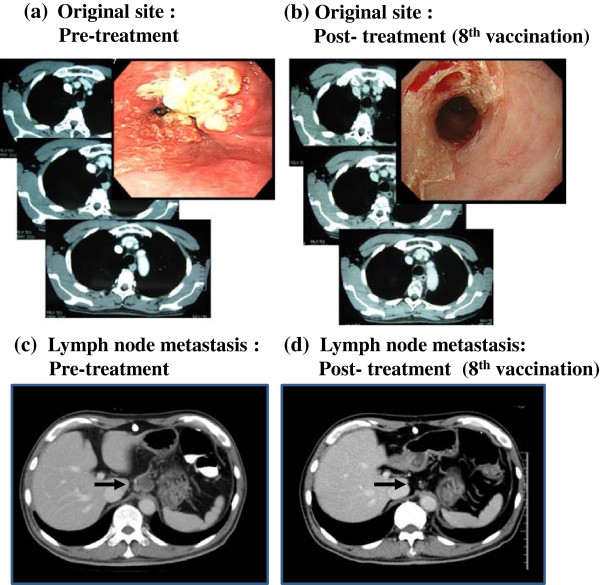
**Computed tomography (CT) and endoscopic images of CR patient (case 6).** The CT and endoscopic images of the original site **(a, b)** and CT images of lymph nodes metastasis **(c, d)** in case 6, before **(a, c)** and after treatment by CRT with 8^th^ vaccination **(b, d)** are shown.

The clinical responses at the time of the 8^th^ peptide vaccination and latest diagnosis points were evaluated according to the RECIST and are summarized in Table 6. At the time of the 8^th^ peptide vaccination in combination with CRT, 6 patients achieved CR (stage II: No. 1, 2, 7; stage III: No.5, 6, 10), and 5 patients revealed PD (stage III: No. 4; stage IV: No. 3, 8, 9, 11). The CR rates were 100% (3/3) in stage II, 75% (3/4) in stage III and 0% (0/4) in stage IV. Furthermore, after that, 6 CR patients were continuously administered the 5 peptide vaccines, once every 2 weeks for 1 year and once a month after that until PD. Five of these 6 patients continued the CR with no evidence of disease for 4.5 years (No. 1), 4.6 years (No. 2), 2.9 years (No. 6), 2.0 years (No.7) and 1.1 years (No.10), and are still alive. Five patients died within 1 year, and 1 patient died within 2 years. Figure 3 shows the representative endoscopic appearance and computed tomography (CT) scan images of the primary cancer and CT scan images of the lymph node in the CR patients (case 6). Complete tumor regression of the primary tumor site (Figure 3c) and lymph node (Figure 3d) were observed after the treatment of the 8^th^ peptide vaccinations with CRT.

**Table 6:** Clinical responses

**Patient no.**	**Stage**	Clinical response*		**Number of vac.**	**CR duration period (year)**	**PFS (days)**	**OS (days)**	**Outcome**
		**8**^**th **^**vac.**	**Final results**					
1	IIA	CR	CR	22	4.5	1659	1775	Alive
2	IIB	CR	CR	85	4.6	1681	1709	Alive
3	IVA	PD	PD	8		67	154	Dead
4	III	PD	PD	8		84	123	Dead
5	III	CR	PD	12		127	765	Dead
6	III	CR	CR	69	2.9	1072	1234	Alive
7	IIB	CR	PD	26	2.0	733	1105	Alive
8	IVB	PD	PD	10		176	231	Dead
9	IVB	PD	PD	8		59	300	Dead
10	III	CR	CR	36	1.1	384	660	Alive
11	IVB	PD	PD	15		155	217	Dead

*Clinical responses of patients were analyzed after the 8^th^ vaccination and the latest diagnosis points. CR: complete response; PD: progressive disease; PFS: Progression-free survival; OS: overall survival.

## Discussion

Our phase I clinical trial comprising a combination therapy of multiple-peptide vaccine and CRT in patients with HLA-A*2402 positive unresectable ESCC proceeded with satisfactory safety levels in all the patients. Peptide-specific CTL could be induced by the all peptide vaccine. In the assessment of clinical response, 6 CR and 5 PD cases were recognized, and 4 CR patients showed a prolonged survival period for 2.0-4.6 years.

The immunotherapies for unresectable or advanced ESCC patients using the peptide vaccines are of interest. Using the NY-ESO-1 peptide mixed with Picibanil OK432 and montanide ISA-51, it has been demonstrated that this peptide vaccine is able to elicite a CD4 and CD8 T cell response [26]. The usefulness of peptide vaccines TTK and URLC10 (LY6K), and KOC1 (IMP-3) have also been investigated and antigen-specific CD8 T cell responses successfully elicited in other studies [22,27]. However, the effects of these studies were limited in their clinical benefit.

In this study, we hypothesized that immunotherapy may achieve effective clearance of residual cancer cells after CRT and that this may improve the prognosis. It had been thought that the main mechanism of the tumor reduction after irradiation is direct damage to tumor DNA by ionizing irradiation. Recently, however, several interesting studies have reported that local irradiation of tumor tissue elicits immunomodulation effects and inhibits tumor growth through the generation of tumor-specific CTL [28]. This concept has been supported by the accumulating data of many investigators based on basic and clinical studies [10-14,29]. Lugade et al. reported that local radiation increased both the generation of the peptide-reactive IFN-γ producing anti-tumor immune cells and their trafficking to the tumor-draining lymph nodes [10]. The essential role of CD8^+^ T-cells in radiation therapy has been demonstrated and it has been suggested that TLR4 on host dendritic cells may be crucial role for induction of these antitumor T cells [11,13,29]. Interestingly, Aktsu et al. demonstrated that radiation itself not only kills the tumor cells but can also evoke a systemic antitumor effect (abscopal effect) by the enhancement of the heat shock protein gp96 [30]. Furthermore, many studies support the immunomodulation effects of chemotherapy. Hattori et al. demonstrated an increase of immunological responses in cases of personalized peptide vaccination combined with UFT and UZEL in metastatic colorectal cancer patients and Sato et al. reported on the usefulness of the personalized peptide vaccine in combination with TS-1 [31,32]. These results suggest that not only radiation but also some kind of anticancer drug may have potential could enhance the anti-tumor response of immunotherapy.

In contrast, clinical trials of immunotherapy combined with CRT have been few in number. Marten et al. evaluated a randomized phase II trial of adjuvant therapy of IFN-α combined with 5-FU/CDDP and radiation in patients with R0 or R1 pancreatic adenocarcinoma, and concluded that this therapy had a minor impact on the clinical response and the lowering of toxicity levels [33]. However, no study has hitherto reported a clinical trial of peptide vaccine combined with CRT in ESCC patients.

In preliminary examination, we investigated the adverse events of CRT standard regimens in Japan, with a focus on the number of lymphocytes which is the key of immunological response of tumor vaccine. Because JCOG9906 showed the less grade of lymphopenia as compared with that of other regimen, we selected this regimen for our study (Additional file 3: Table S2). In our clinical trial, we have demonstrated that our protocol was well-tolerated. Major toxicities were myelo-suppression and esophagitis may be related to CRT, and grade 3 toxicities of leucopenia, neutropenia, anemia and thrombocutopenia occurred in 54.5%, 27.3%, 27.3% and 9.1% of patients, respectively. With regards to the toxicities of unresectable advanced ESCC patients treated with CRT, it was reported that grade 3 leukopenia, neutropenia, anemia and esophagitis were observed in 33.3%, 8.3%, 6.7% and 3.3% of the patients, respectively, and grade 4 platelet, dyspnea and infection were observed in 3.3%, 1.7%, and 1.7% of the patients, respectively [8]. In contrast to these studies, our protocol did not show any grade 4 hematological toxicity and non-hematological toxicity. As toxicity related to peptide vaccine, grade 1 local skin reactions at the injection sites of peptide vaccines were observed in 81.8% of patients, which is almost the same results with that of previous study of peptide vaccines for ESCC patients [25].

In the guidance of FDA for cancer vaccine, it states that the maximum tolerated dose for a cancer vaccine may not be identified except in very rare situations. Furthermore it was also described that, when no DLT is expected or achieved, optimization of other outcomes, such as the immune response, can be useful to identify doses for subsequent studies [34]. In our study, DLT was not detected in any patients. However, in a comparison of the CTL induction of 8th vaccination, we observed that the 3 mg of peptide seems to induce peptide-specific CTLs more effectively than the 0.5 mg and 1 mg levels. Therefore, we recommend that 3 mg be the basis for future study.

**Figure 4:**
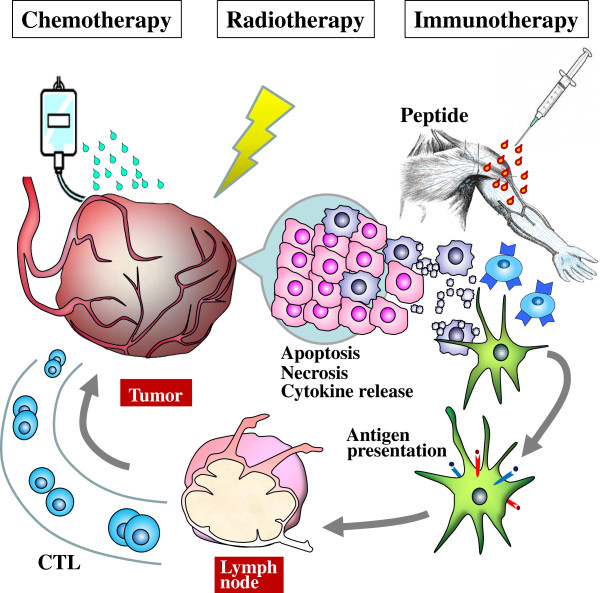
Mechanism of peptide vaccine therapy combined with CRT.

In this study, all biopsy samples of ESCC tissues collected from patients showed clear expressions of HLA class I antigen and all peptides antigens (URLC10, TTK, KOC1, VEGFR1, and VEGFR2). Positive rates of peptide specific CTL reactions at the 8^th^ vaccination were 54.5% for URLC10, 36.4% for TTK, 45.5% for KOC1, 45.5% for VEGFR1 and 36.4% for VEGFR2, and then increased to 40-100% at the 16^th^ vaccination. CTL positive levels for URLC10, TTK and KOC1 are almost the same as those found in previous reports which used the peptide vaccine in ESCC patients [22,23]. Regarding the mechanism of CTL production, we speculate that the apoptosis and necrosis of cancer cells by CRT may induce a pro-inflammatory response in the tumor environment, and may enhance the immunogenicity of the cancer cells and antigen presentation of DC, and that this may result in increased CTL production (Figure 4).

The clinical responses in our study were 6 CR (3 patients with stage II, 3 patients with stage III) and 5 PD (1 patient with stage III, 4 patients with stage IV). It has been reported that CRT itself showed CR at a rate of 62% (78% in the T1-2 disease and 55% in the T3 disease) [7]. However, it is known that 30-50% of patients treated with CRT recur within 1 year [4,5]. In contrast, our study showed that in 4 cases out of 6 CR patients showed a long and continuous CR period (2.0-4.6 years). This study is a phase I trial and the primary endpoint was toxicity. The patient number of this study is small and we did not include the CRT patients without peptide vaccines as a comparison group. Therefore, it is difficult to clarify the clinical significance of these prolonged CR periods, at this stage. We expect that anti-tumor effect of peptide vaccine for residual cancer cells after CRT will become apparent in phase II trials.

## Conclusions

To the best of our knowledge our study is the first clinical trial of a multi-peptide vaccine combined with CRT that has demonstrated the safety and induction of peptide specific CTL followed by a long CR period in unresectable ESCC patients. Our results point to the need to move forward to the next-stage of this combination therapy in an adjuvant setting of ESCC patients.

## Abbreviations

ESCC: Esophageal squamous cell carcinoma; CRT: Chemoradiation therapy; TTK: TTK protein kinase; URLC10: Up-regulated lung cancer 10; KOC1: Insulin-like growth factor–II mRNA binding protein 3; VEGFR1: Vascular endothelial growth factor receptor 1; VEGFR2: Vascular endothelial growth factor receptor 2; ELISPOT: Enzyme-linked immunospot; PBMC: Peripheral blood mononuclear cells; CTL: Cytotoxic T lymphocytes; CR: Complete clinical response; PD: Progressive disease; IHC: Immunohistochemical; PFS: Progression free survival.

## Competing interests

K. Yoshida and T. Tsunoda are current employed (other than primary affiliation; e.g., consulting) by OncoTherapy Science, Inc. Y. Nakamura is a stockholder and scientific advisor for OncoTherapy Science, Inc. No potential conflicts of interest were disclosed by the other authors.

## Authors’ contributions

HI participated in the conception, design and the writing of manuscript. RF and KO participated in the design, review and revision of the manuscript. TI, JT and MH participated in the analysis and interpretation of data. KT, KY and TT participated in the immunological assay. All authors participated in the data acquisition and discussion of the manuscript. All authors read and approved the final manuscript.

## Supplementary Material

Additional file 1: Figure S1 Treatment protocol. Click here for file

Additional file 2: Table S1 Peptide antigen-specific CTL responses in PBLs evaluated by ELISPOT assay. Click here for file

Additional file 3: Table S2 Comparison of toxicities in two regimens of CRT. Click here for file
