# Supplementary figures and images for: Phase I clinical study of multiple epitope peptide vaccine combined with chemoradiation therapy in esophageal cancer patients
Source: J Transl Med. 2014 Apr 3;12:84. doi: 10.1186/1479-5876-12-84 (PMC4234129; doi:10.1186/1479-5876-12-84)

Additional file 1

Figure S1: Treatment protocol

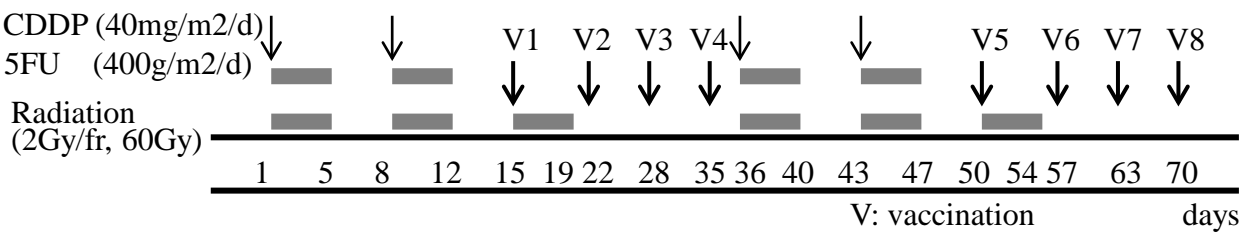

Supplement: Additional file 1: Figure S1 — Treatment protocol. [file 1479-5876-12-84-S1.pdf]
