# Supplementary material for: Phase I clinical study of multiple epitope peptide vaccine combined with chemoradiation therapy in esophageal cancer patients
Source: J Transl Med. 2014 Apr 3;12:84. doi: 10.1186/1479-5876-12-84 (PMC4234129; doi:10.1186/1479-5876-12-84)
Supplement: Additional file 2: Table S1 — Peptide antigen-specific CTL responses in PBLs evaluated by ELISPOT assay. [file 1479-5876-12-84-S2.docx]

Additional file 2

Table S1. Peptide antigen-specific CTL responses in PBLs evaluated by ELISPOT assay

| No. | Peptide  (mg) | Number  of vac. | CTL activity* | | | | | |
| --- | --- | --- | --- | --- | --- | --- | --- | --- |
|  |  |  | URLC10 | TTK | KOC1 | VEGFR1 | VEGFR2 | Positive Cont.(CMV) |
| 1 | 0.5 | Pre | － | ＋ | － | － | － | ＋＋＋ |
|  |  | 4 | ＋ | ＋ | － | － | ＋ | ＋＋＋ |
|  |  | 8 | － | － | － | － | － | ＋＋＋ |
|  |  | 12 | ＋ | － | ＋ | － | ＋＋ | ＋＋＋ |
|  |  | 16 | ＋＋＋ | ＋＋＋ | ＋ | ＋＋ | ＋ | ＋＋＋ |
| 2 | 0.5 | Pre | ＋ | － | ＋ | － | ＋ | ＋＋＋ |
|  |  | 4 | ＋ | － | ＋ | ＋ | － | ＋＋＋ |
|  |  | 8 | － | － | － | ＋ | ＋ | ＋＋＋ |
|  |  | 12 | ＋ | ＋ | ＋ | NA | － | ＋＋＋ |
|  |  | 16 | ＋＋＋ | － | － | ＋ | － | ＋＋＋ |
| 3 | 0.5 | Pre | － | － | － | － | － | ＋＋＋ |
|  |  | 4 | ＋＋ | － | － | ＋ | － | ＋＋＋ |
|  |  | 8 | － | － | － | － | － | ＋＋ |
| 4 | 1 | Pre | － | － | － | ＋ | － | ＋＋＋ |
|  |  | 4 | ＋ | － | ＋ | ＋＋ | ＋ | ＋＋＋ |
|  |  | 8 | ＋＋＋ | － | － | － | NA | ＋＋＋ |
| 5 | 1 | Pre | － | ＋ | ＋ | NA | NA | ＋＋＋ |
|  |  | 4 | － | － | － | ＋ | － | ＋＋＋ |
|  |  | 8 | － | － | － | ＋ | － | ＋＋＋ |
| 6 | 1 | Pre | － | － | － | － | ＋ | ＋＋＋ |
|  |  | 4 | － | － | － | NA | ＋ | ＋＋＋ |
|  |  | 8 | ＋＋ | ＋ | － | － | － | ＋＋＋ |
|  |  | 12 | ＋＋ | － | － | － | ＋ | ＋＋＋ |
|  |  | 16 | ＋＋＋ | － | － | － | ＋ | ＋＋＋ |
| 7 | 3 | Pre | － | － | NA | － | － | ＋＋＋ |
|  |  | 4 | NA | － | － | NA | － | ＋＋＋ |
|  |  | 8 | － | － | ＋ | － | － | ＋＋＋ |
|  |  | 12 | ＋ | － | － | ＋ | ＋ | ＋＋＋ |
|  |  | 16 | ＋＋＋ | － | ＋ | － | ＋ | ＋＋＋ |
| 8 | 3 | Pre | － | － | － | － | － | ＋＋＋ |
|  |  | 4 | ＋＋＋ | － | － | － | － | ＋＋＋ |
|  |  | 8 | ＋＋＋ | ＋＋＋ | ＋ | － | － | ＋＋＋ |
| 9 | 3 | Pre | － | － | － | － | － | ＋＋＋ |
|  |  | 4 | ＋ | － | ＋ | － | － | ＋＋＋ |
|  |  | 8 | ＋＋＋ | － | ＋ | ＋ | ＋ | ＋＋＋ |
| 10 | 3 | Pre | ＋＋ | ＋ | ＋ | ＋ | ＋ | ＋＋＋ |
|  |  | 4 | ＋ | ＋ | ＋ | ＋ | ＋ | ＋＋＋ |
|  |  | 8 | ＋＋ | ＋＋ | ＋ | ＋＋ | ＋ | ＋＋＋ |
|  |  | 12 | ＋＋＋ | ＋ | ＋＋ | ＋ | ＋ | ＋＋＋ |
|  |  | 16 | ＋＋＋ | ＋＋ | ＋＋ | ＋ | ＋＋ | ＋＋＋ |
| 11 | 3 | Pre | ＋ | ＋ | ＋ | － | ＋ | ＋＋＋ |
|  |  | 4 | － | ＋ | ＋ | － | ＋ | ＋＋＋ |
|  |  | 8 | ＋ | ＋ | ＋ | － | ＋ | ＋＋＋ |

*The positivity of the antigen-specific T cell responses was classified into four grades (-, +, ++ and +++), depending on the peptide-specific spots at different responder/stimulator ratios.

NA: These data were not analyzed because of the low number of cells (<45000).
