# Supplementary material for: Phase I clinical study of multiple epitope peptide vaccine combined with chemoradiation therapy in esophageal cancer patients
Source: J Transl Med. 2014 Apr 3;12:84. doi: 10.1186/1479-5876-12-84 (PMC4234129; doi:10.1186/1479-5876-12-84)
Supplement: Additional file 3: Table S2 — Comparison of toxicities in two regimens of CRT. [file 1479-5876-12-84-S3.docx]

Additional file 3

Table S2. Comparison of toxicities in two regimens of CRT

(JCOG9906, JCOG9516)*

| Case No. | Regimen of CRT | Grade of  leukopenia | Grade of  lymphopenia |
| --- | --- | --- | --- |
| 1 | JCOG9906 | 0 | 2 |
| 2 | JCOG9906 | 3 | 3 |
| 3 | JCOG9906 | 2 | 3 |
| 4 | JCOG9906 | 2 | 3 |
| 5 | JCOG9906 | 1 | 3 |
| 6 | JCOG9516 | 2 | 3 |
| 7 | JCOG9516 | 0 | 3 |
| 8 | JCOG9516 | 3 | 4 |
| 9 | JCOG9516 | 2 | 3 |
| 10 | JCOG9516 | 3 | 4 |

*Treatment protocol of JCOG9906 and JCOG9516 regimens are written in Ref 7,

and Ref 8, respectively.
